# Effect of Echinacea on gut microbiota of immunosuppressed ducks

**DOI:** 10.3389/fmicb.2022.1091116

**Published:** 2023-01-05

**Authors:** Renzhao Lin, Chanping Zhi, Yalin Su, Jiaxin Chen, Debao Gao, Sihan Li, Dayou Shi

**Affiliations:** ^1^College of Veterinary Medicine, South China Agricultural University, Guangzhou, China; ^2^Guangdong Maoming Agriculture and Forestry Technical College, Maoming, China; ^3^Guangzhou Technician College, Guangzhou, China

**Keywords:** gut microbiota, immunosuppression, Echinacea extract, duck, *Prevotella*

## Abstract

**Introduction:**

Immunosuppression puts animals in a susceptible state and disrupts the balance of intestinal flora, which can increase the risk of disease and cause serious harm to the farm. Echinacea can exert its immunomodulatory effect in various ways, but its influence on intestinal flora is unclear.

**Methods:**

Therefore, we investigated the effect of Echinacea extract (EE) on gut microbiota in immunosuppressed ducks by 16s-RNA sequencing in this experiment.

**Results:**

The results showed that EE significantly improved the weight gain of immunosuppressed ducks (*p*<0.001). It also increased the immune organ index (*p*<0.01) and upregulated the levels of TNF-α and IFN-γ (*p*<0.05) as well as IL-2 in the serum. The lesions of the bursa were evident compared to the spleen and thymus. After treatment in the EE group, the lymphocyte count of the bursa returned to healthy levels and the lesions were significantly improved. The diversity analysis showed that neither of the alpha-diversity indices showed a significant difference (*p*>0.05). However, the EE group had a trend closer to the healthy group compared to the M group. β-diversity analysis revealed a high degree of sample separation between the healthy and immunosuppressed groups. The sequencing result showed a significantly higher relative abundance of *Prevotella* and *Prevotella_UCG_001* in the dexamethasone-treated group, which could be potential biomarkers of dexamethasone-induced immunosuppression. EE increased the relative abundance of *Akkermansia*, *Bacteroides*, and *Alistipes* and significantly decreased the relative abundance of *Megamonas*, *Streptococcus*, and *Enterococcus* (*p*<0.05).

**Conclusion:**

The results showed that Echinacea extract improves the development of immunosuppressed ducks and modulates intestinal immune function by increasing the abundance of beneficial bacterial genera in the intestine.

## 1. Introduction

China is the world’s largest producer and consumer of waterfowl, including the meat duck, egg duck, and meat goose industries. The total value of waterfowl production has exceeded $100 billion, with duck farming accounting for 74.3% of world production and goose farming for 93.3%. They can provide large quantities of high-quality meat and down. In recent years, diseases caused by immunosuppression have become more and more prevalent in large intensive farms, and the direct or indirect losses and hazards caused by them are quite huge. Immunosuppression can lead to retarded weight gain, decreased egg production in laying hens, and decreased litter size in breeding pigs by affecting animal intake and reducing feed conversion ratios. Meanwhile, the animals are vulnerable to infection, erosion by pathogenic microorganisms, and secondary diseases, which can be fatal in serious cases. However, there is still a gap in studies related to immunosuppression in waterfowl compared to reports in chickens, pigs, and rats. The factors leading to immunosuppression are mainly divided into disease factors, human factors, and feeding environment factors. Most of the factors causing immunosuppression in ducks are viral diseases, such as duck circovirus (DCV) (Hong et al., 2018), duck euthero virus (Wang et al., 2020), duck influenza virus, duck herpesvirus type 2, duck distemper virus (DPV) (Dhama et al., 2017), etc. These diseases are characterized by damage to the immune organs and hinder the process of the humoral immune response.

Dexamethasone can cause an immunosuppressive state in animals, and it was selected as an immunosuppressive drug in this test. In experiments studying animal models of dexamethasone-induced immunosuppression pathology, more attention has been paid to changes in leukocytes and immune cells, and a lack of focus on clinical signs such as body weight (Lo et al., 2005; Harada et al., 2011; Hundakova et al., 2022). Immunosuppression led to atrophy of the thymus and the bursin, organ function was affected, and organ indexes showed a significant decrease after modeling, whereas the spleen showed no difference. It was found that dexamethasone-induced immunosuppression significantly reduced splenic lymphoid follicles in the spleen of house sparrows. But did not affect their CD3 immune effect and had a minimal effect on splenic lymphocytes in mice (Jeklova et al., 2008; Crouch et al., 2022).

Research on natural herbal medicines is critical to reducing the risk of drug resistance on farms. Echinacea, as a natural herb, possesses a wide range of medicinal effects, and it contains a great potential medical value that is worth exploring. Echinacea was already used to treat traumatic injuries, septicemia, and toothache by Indians in the 18th century. Nowadays, it is more commonly used to treat skin diseases and to combat respiratory diseases such as influenza and asthma in Western countries (Aarland et al., 2017). A large number of studies have also reported that EE can exert immunomodulatory effects by affecting immune system mechanisms in different ways (Block and Mead, 2003; Randolph et al., 2003; Sharifi-Rad et al., 2018), such as activating immune cells and promoting the secretion of interferon-α (Zhai et al., 2007). However, the effects of its interaction with the intestinal flora on the immune system are still inconclusive.

It has been found that the immune regulation of the body is inseparably related to gut microbiota (Hansen et al., 2010). The gut microbiota is a system composed of a large variety of bacteria, including beneficial, harmful, and neutral bacteria. These microbiotas play a key role in digestion and absorption, growth and development, immune regulation, and physiological and structural changes in the intestine (Liu et al., 2009; Quinteiro-Filho et al., 2012). The immune function of the host is closely linked to the dynamic balance of the gut microbiota (Yamashiro, 2017; Liu et al., 2021). Normal flora has an important role in promoting the maturation of immune cells and tissues, while the presence of imbalances in the flora, is associated with the development of infectious and inflammatory diseases such as bacterial vaginosis, inflammatory bowel disease, and rheumatoid arthritis (Srinivasan et al., 2012; Ferreira et al., 2014; Trompette et al., 2014; Wagenaar et al., 2021). The gut microbiota can affect the host’s immune system in direct or indirect ways. The flora directly eradicates pathogenic competitors by competing for nutrients and ecological niches, acting as a biological barrier together with the intestinal mucosa; or indirectly influencing the host’s immune system through flora metabolites, enhancing its defense mechanisms (Kamada et al., 2013). For example, SCFAs are common metabolites of the flora, mainly produced by Firmicutes and Bacteroidota. They provide energy to intestinal epithelial cells, maintain the integrity of the intestinal mucosa, balance the pH of the intestinal microenvironment, have a positive regulatory effect on intestinal immune cells, and exert an inhibitory effect on intestinal inflammation (Correa-Oliveira et al., 2016; Parada et al., 2019; Blaak et al., 2020).

Abnormalities in the species, ratio, and the number of gut microbiota could occur due to medical origin, drug abuse, and other problems. The immune regulation and metabolic function of gut microbiota will be affected as the homeostasis of the microbial population are out of order. As a result, changes in the intestinal flora may lead to disruption of the normal immune response process and even immunosuppression. It may also lead to changes in the microenvironment in the intestinal tract and abnormalities in the digestive and absorption functions of the animal. This effect can affect the increase in body weight, decrease in meat yield, increase in feed weight ratio, etc., causing economic losses to the farm (Choi et al., 2014).

In this experiment, we analyzed the effect of EE on the treatment of the dexamethasone immunosuppressed duck model by the 16 s-RNA intestinal flora sequencing method and explored the relationship between the immunomodulatory effect of EE and intestinal flora.

## 2. Materials and methods

### 2.1. Animals and treatment

The protocol was performed after the approval of the Institutional Animal Welfare and Research Ethics Committee of South China Agricultural University, Guangzhou, China, and every effort was made to minimize animals suffering during the experiments. A total of 60 healthy 7-day-old Pekin ducks (purchased from Foshan Guiliu Poultry Co., Ltd.) were randomly divided into three groups of 20 ducks each. They were divided into a blank group (K), a model control group (M) and an Echinacea extract treatment group (EE). In the M and EE groups, dexamethasone (purchased from Chongqing Buur Animal Pharmaceutical Co., Ltd.) was injected intramuscularly at a dose of 3.5 mg/kg for 7 days to construct an immunosuppressed animal model, with no dexamethasone injection in group K. After the animal model was established, the EE group added 0.6 g/kg of Echinacea purpurea extract powder (purchased from Sichuan Hengrui Tongda Veterinary Medicine Co., Ltd.) to the basic diet, while the K and M groups had no addition to the basic diet. During the experiment, all three groups were fed and watered *ad libitum*.

### 2.2. Body weight, immune organ index, and serum cytokines

We randomly selected six ducks from each group and sampled them at 0, 7, and 14 days after EE administration. The ducks were euthanized. And the weight of the body, spleen, thymus, and bursa of each duck was measured and recorded.

The immune organ index is calculated as follows. Immune organ index = immune organ weight (mg)/body weight (g). Their blood was obtained from the jugular vein, centrifuged at 3000 rpm/min for 10 min, and the serum was collected to detect the TNF-α, IFN-γ, and IL-2 levels in it by Elisa.

### 2.3. Pathological histological sections

After modeling, the spleen, thymus, and bursa of ducks in the healthy and immunosuppressed groups were randomly dissected and placed in 10% neutral formalin fixation, paraffin-embedded and HE stained to observe histopathology. According to the pathological changes, test ducks were randomly selected for dissection at 7 and 14 days of treatment and immune organs with lesions were obtained for HE staining to observe the pathological changes.

### 2.4. Study on the diversity of cecum contents microbiota

After dissection of 5 randomly selected test ducks in each group at 14 d after the administration, 2 g of cecum contents were placed in lyophilized tubes and stored at −80°C for the study of intestinal contents flora diversity. The total genomic DNA of the samples was extracted by CTAB/SDS method, and the DNA concentration and purity were detected on 1% agarose gel. Depending on the concentration, DNA was diluted to 1 ng/μL with sterile water, and the 16S rRNA genes of different regions were amplified with specific primers and barcodes. Equal amounts of 1X loading buffer (containing SYB green) were mixed with PCR products, DNA detection was performed on a 2% agarose gel, and the mixed PCR products were purified using Qiagen Gel Extraction Kit. Sequencing libraries were generated using the NEBNext® Ultra™ IIDNA Library Prep Kit (Cat No. E7645). Library quality was assessed by Qubit@2.0 fluorometer (Thermo Science) and Agilent Bioanalyzer 2,100 system. Finally, the library was sequenced on the Illumina NovaSeq platform and a 250 bp paired-end read was generated.

To continue expanding the sequencing volume, the sample size was first predicted and measured by plotting sparsity and species accumulation curves. Based on the results of species annotation, the top 10 species with maximum abundance in each group from taxonomic levels of phylum and genus were selected to generate cumulative bar charts of species relative abundance to visualize species with greater relative abundance at different taxonomic levels and their proportions. Alpha diversity reflects the richness of the sample communities through Chao1, Dominance, Observed_otus, Pielou_e, Shannon, and Simpson. Beta diversity was analyzed by PCA for similarity and similarity in the community structure of different samples. The top 35 genera in terms of abundance were selected and clustered at both species and sample levels based on species annotation and abundance information and plotted as a heat map to facilitate the discovery of the high and low aggregation content of species in each sample. Species abundance data between groups were hypothesis tested using the MetaStat method to obtain *p*-values, species with significant differences between groups were screened based on p-values, and histograms of differential species between groups were plotted. To discover and interpret high-dimensional biomarkers (genes, pathways, and taxonomic units), comparisons were performed using the LEfSe (LDA Effect Size) analysis tool (Segata et al., 2011) to find statistically different Biomarkers between groups based on statistical significance and biological relevance. In addition, KO database-based metabolic function prediction of the colony was performed by PICRUSt2 based on 16S sequencing data.

### 2.5. Data statistical analysis

The raw data of each group was collected during the experiment and analyzed by IBM SPSS Statistics 26 statistical analysis software. The values were analyzed with One-way ANOVA, LSD, and Kruskal-Wallis tests and converted to graphs by GraphPad Prism 8. The analysis results are expressed as “mean ± standard error.”

## 3. Results

### 3.1. Effect of Echinacea on growth performance and immune enhancement

**Figure 1:**
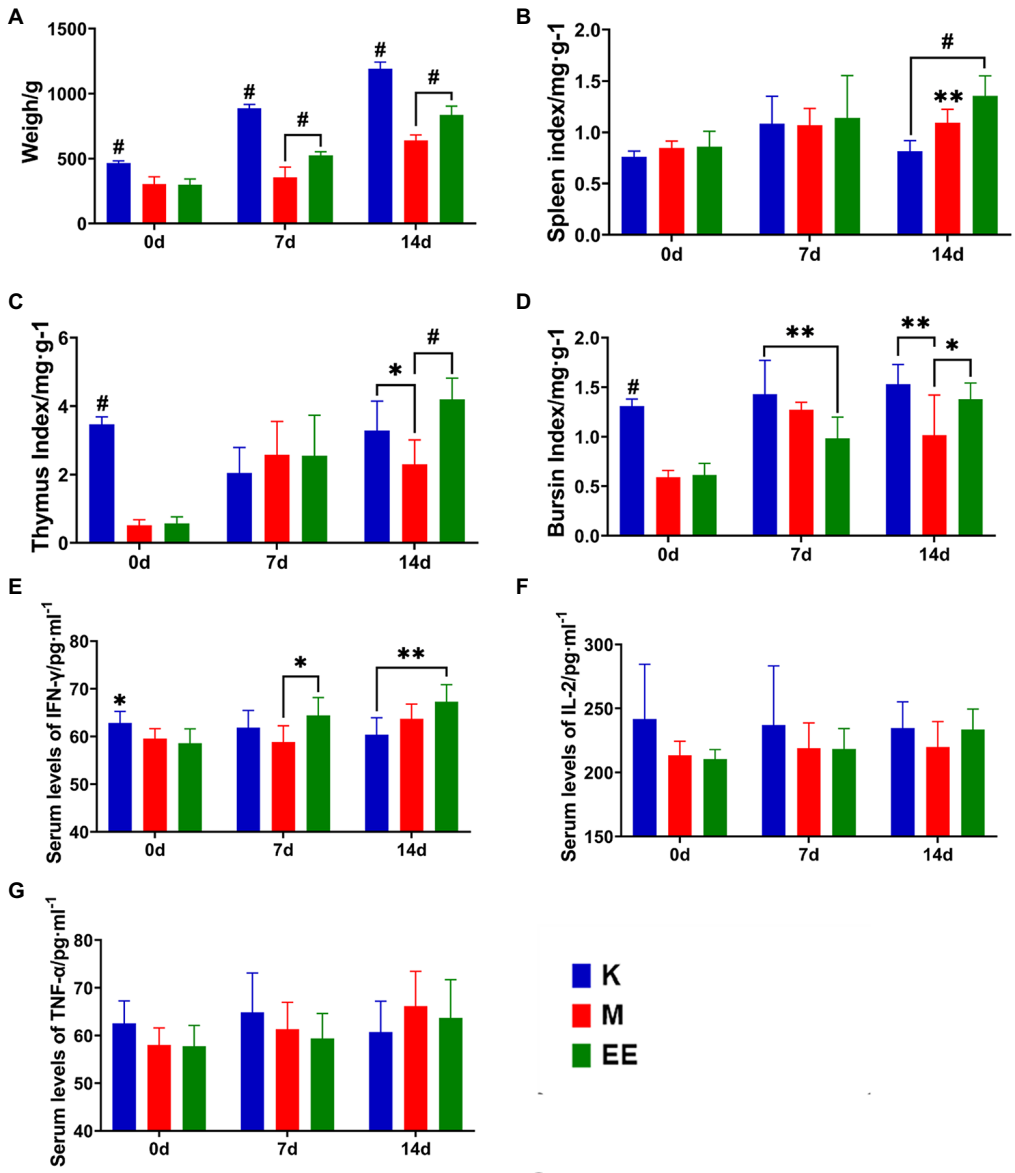
Effect of Echinacea on growth performance and immune enhancement. **(A)** Body weight; **(B–D)** Immune organ indices; **(E–G)** Levels of cytokine content in serum. *N* = 6, *p* < 0.05 (*), *p* < 0.01 (**), *p* < 0.001 (#).

The results showed that Echinacea extract significantly improved the slow body weight gain and decreased immune organ index levels caused by immunosuppression, and increased the levels of IFN-γ, TNF-α, and IL-2 in the serum of immunosuppressed ducks. The immunosuppressed animal model was established after 7 days of continuous dexamethasone injection. The body weight of animals in the immunosuppressed group was significantly lower compared to the K group (*p* < 0.001). Echinacea extract was started in the EE and M groups. At 7 and 14 days, the body weight of ducks in the EE group was significantly higher than in the M group (*p* < 0.001). However, there was still a significant difference compared to the K group (*p* < 0.001) (Figure 1A). In the comparison of immune organ indices between the groups, the spleen index showed a significant difference between the EE and M groups only at 14 days of the administration, with the EE group being significantly higher than the M group (*p* < 0.01) (Figure 1B). While before treatment with Echinacea extract, the thymic and bursal indices showed significant differences between the healthy control group and the immunosuppressed group, immunosuppression significantly reduced the levels of both of these immune organ indices (*p* < 0.001). At 14 days of the administration, the EE group showed a significant recovery in the thymus (*p* < 0.001) and bursal (*p* < 0.05) organ index levels, both higher than the M group (Figures 1C,D). The levels of IFN-γ, TNF-α, and IL-2 in the serum of the EE group showed a tendency to increase during drug administration (Figures 1E–G). IFN-γ showed a significant decrease (*p* < 0.05) after immunosuppression. But at 7 days of drug administration, it was significantly higher (*p* < 0.05) in the EE group compared with the M group (Figure 1E).

### 3.2. Effect of Echinacea on histopathology of immune organs

**Figure 2:**
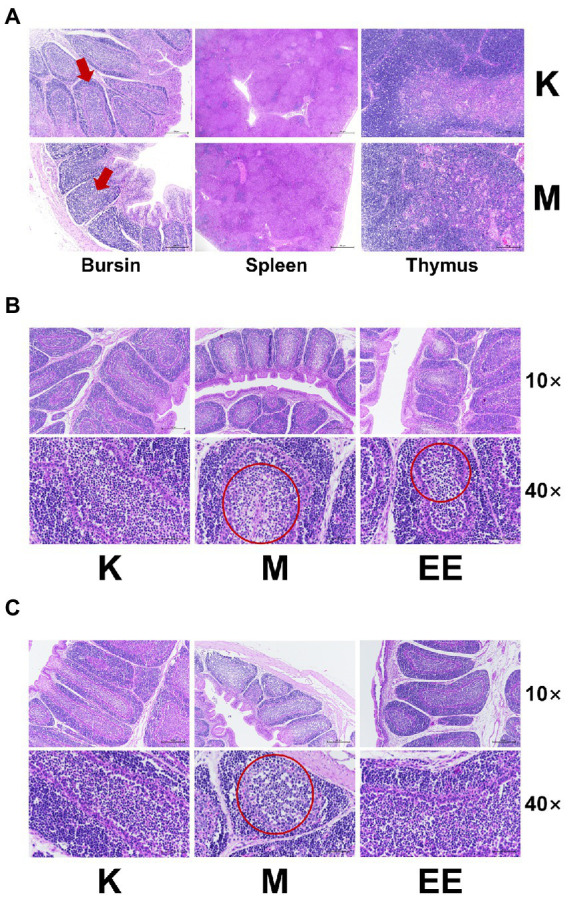
Effect of Echinacea on histopathology of immune organs (HE staining). **(A)** Pathological changes of immune organs after 7 days of immunosuppression. The area indicated by the red arrow shows a poorly demarcated bursal skin medulla; **(B)** Pathological changes of bursa of each group after 7 days of treatment; **(C)** Pathological changes of bursa of each group after 14 days of treatment. In the area circled by the red circle, the cells are less aggregated and sparser and the number of lymphocytes is reduced. (*N* = 6).

After modeling, the cortical and medullary boundaries of the lymph nodules of the bursa phalloides in the model group were indistinct, the epithelial reticular cell layer disappeared, and the cortical and medullary lymphocytes were significantly reduced. In contrast, the spleen and thymus showed no significant abnormalities (Figure 2A). Therefore, after the administration, the bursa was taken for staining at 7 and 14 days, respectively. In the bursa of group K, the lymph nodes were demarcated between the cortex and the medulla, separated by epithelial reticular cells, and there were a large number of lymphocytes in the cortex and medulla. In contrast, lymphocytes were significantly reduced in the M group and slightly reduced in the EE group after 7 days of treatment (Figure 2B). After 14 days, lymphocytes in the cortex and medulla of the bursa of the M group decreased significantly, medullary lymphocytes showed vacuolar degeneration, while the number of lymphocytes in the EE group recovered to healthy levels (Figure 2C).

### 3.3. Regulation of gut flora abundance in immunosuppressed ducks by Echinacea purpurea

The number of species that could be observed leveled off when the sample size reached 19–20, showing that the depth and richness of this sequencing test could already indicate the diversity of species in the sample community. The sequencing results are reliable and can be used for subsequent data analysis.

**Figure 3:**
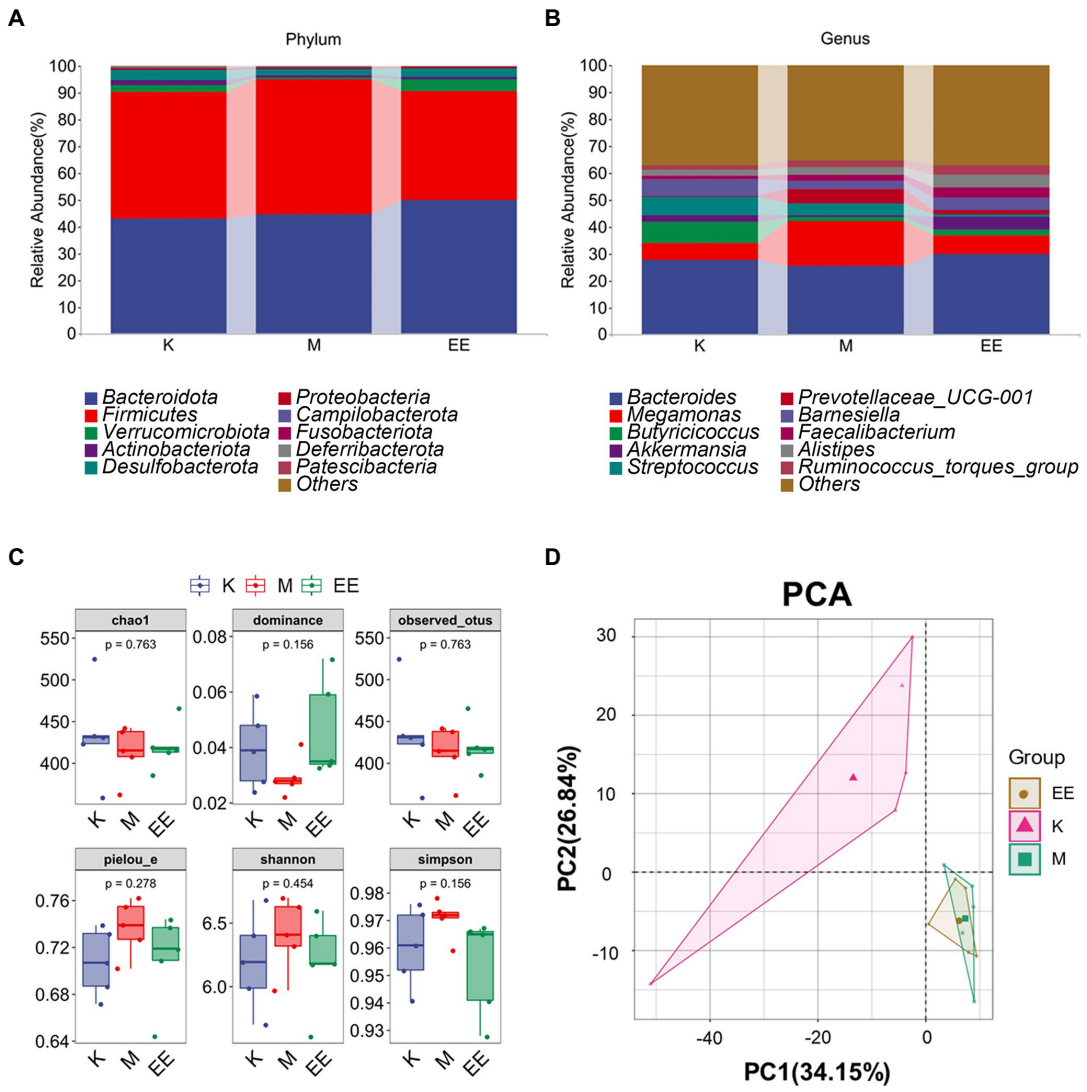
Regulation of gut flora abundance in immunosuppressed ducks by Echinacea purpurea. **(A)** Cumulative histogram of the top 10 species in relative abundance at the phylum taxonomic level; **(B)** Cumulative histogram of the top 10 species in relative abundance at the genus taxonomic level; **(C)** Box plot of the alpha diversity index; **(D)** PCA analysis of beta diversity. (*N* = 5).

Among the components of the gut microbial community at the phylum taxonomic level in each group of ducks, *Bacteroidota*, *Firmicutes*, *Desulfobacterota*, *Actinobacteriota*, and *Verrucomicrobiota* were the main dominant microbiotas. The species composition of the K and EE groups was similar, with *Bacteroidota*, *Firmicutes*, and *Verrucomicrobiota* as the main dominant microbiotas. The relative abundance of *Bacteroidota* increased to 49.86% in the EE group, which was markedly higher compared to the K (43.14%) and M (44.46%) groups. The relative abundance of Firmicutes was significantly lower in the EE group (40.68%) compared to the K group (47.19%) and the M group (50.58%). The relative abundance of *Verrucomicrobiota* in the EE group reached 4.55%, more than that of the K group (2.59%) and the M group (0.67%) (Figure 3A). At the genus classification level, *Bacteroides*, *Butyricicoccus*, *Akkermansia*, *Megamonas*, and *Streptococcus* are the main dominant microbiotas. The relative abundance of *Bacteroides* in the EE group (29.88%) is more than that of the K (27.62%) and M (25.51%) groups. The relative abundance of *Megamonas* markedly increased in the M group (16.51%) compared to the K (6.36%) and EE (6.68%) groups. The relative abundance of *Akkermansia* in the EE group reached 4.55%, more than that of the K (2.59%) and M (0.67%) groups. Remarkably, the relative abundance of *Prevotellaceae_UCG-001* in the M group was up to 5.34%, while that of the K group was only 0.25%, and the EE group was 1.36% (Figure 3B).

### 3.4. Effect of Echinacea on the diversity of intestinal flora

None of the α-diversity indices showed significant differences (*p* > 0.05). But the EE group showed a trend of recovery in all indexes compared to the M group. The indices of Chao1, Dominance, and Observed_otus in the M group were lower than those of the K group. In contrast, the indices of the EE group were closer to the K group than the M group. The Shannon, Simpson, and Pielou_e indices increased in group M compared to group K, but those in group EE decreased to a similar level to group K compared to group M (Figure 3C). Analysis of β-diversity using PCA revealed a significant degree of sample separation between the healthy and immunosuppressed groups and a marked effect of immunosuppression on the gut microbiota. The EE group was more similar to the M group, indicating that no significant changes in the diversity of the gut microbial community were produced after the administration (Figure 3D).

### 3.5. Clustering of the main intestinal flora affected by Echinacea

**Figure 4:**
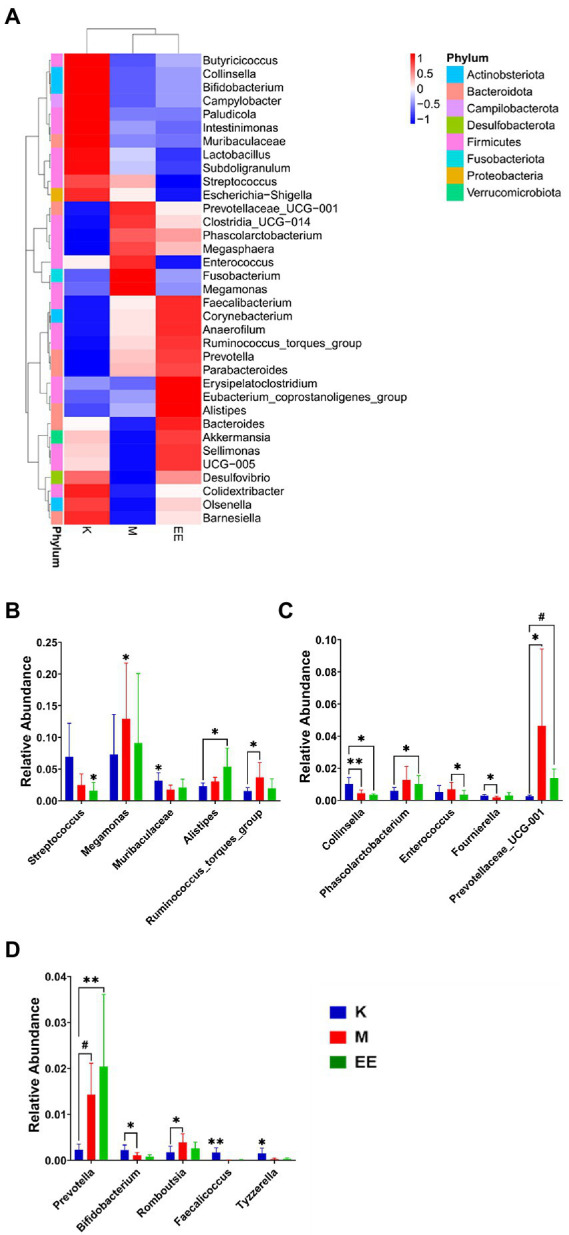
Species abundance clustering analysis and analysis of species differences between groups. **(A)** Clusters of species abundance in the top 35 relative abundances at the genus level; **(B–D)** Histograms of species abundance with significant differences between groups (top 15). *N* = 5, *p* < 0.05 (*), *p* < 0.01 (**), *p* < 0.001 (#).

The top 35 genera in terms of abundance were selected and clustered at both species and sample levels. There were 19 genera belonging to *Firmicutes* and seven genera belonging to *Bacteroidota*. The genera that showed differences in variation due to immunosuppressive effects were mainly in these two groups. It can also be found that the abundance of some genera in the EE group is more convergent to the healthy group compared to the M group. *Enterococcus*, *Megamonas*, and *Fusobacterium* were more abundantly aggregated in the M group, while the genera with higher abundance aggregation in the EE group included *Akkermansia*, *Bacteroides*, and *Alistipes* (Figure 4A).

### 3.6. Analysis of species differences between groups

**Figure 5:**
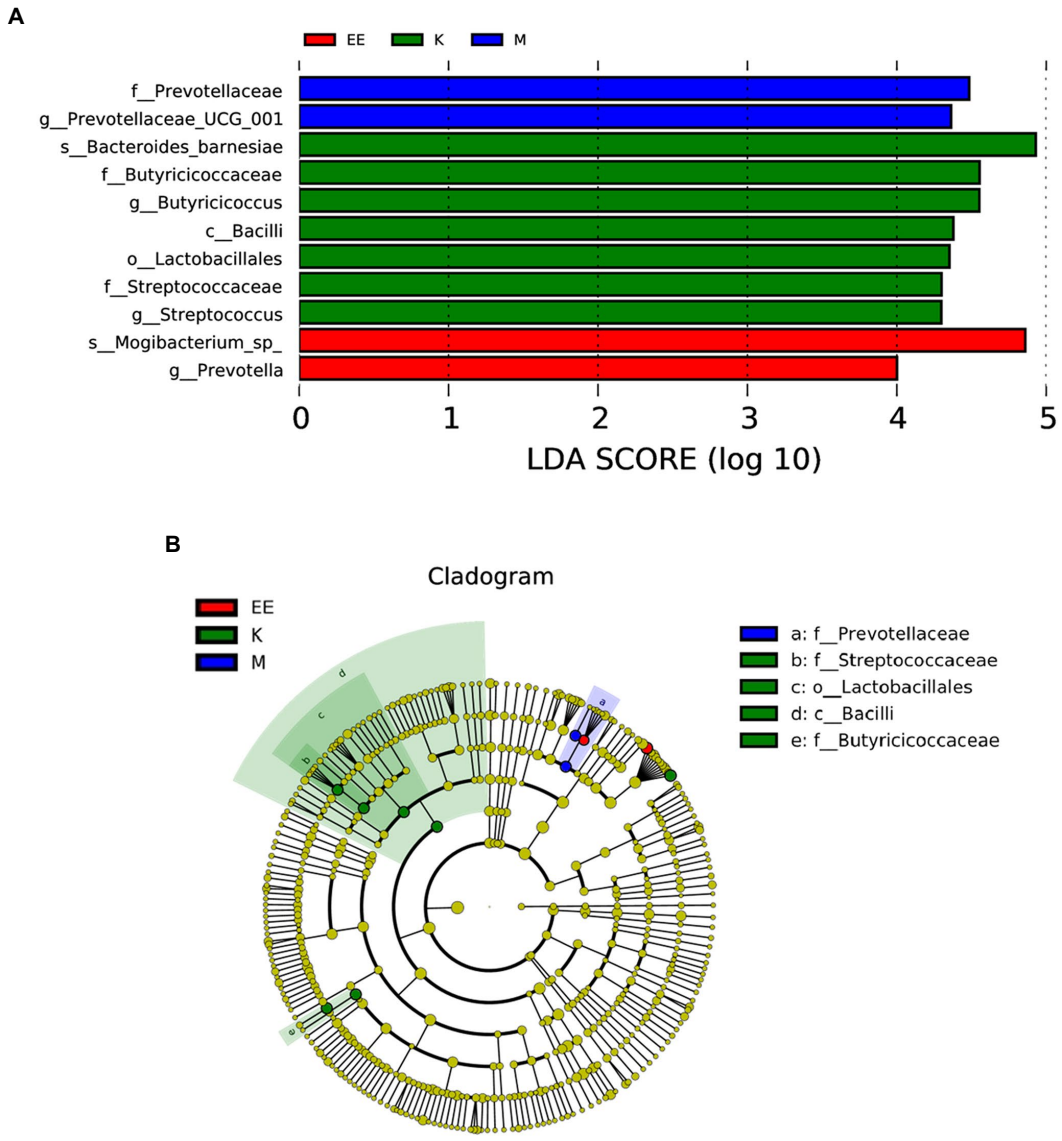
Analysis of species differences between groups. **(A)** Histogram of the distribution of LDA values; **(B)** Evolutionary branching plots, *N* = 5. Species with LDA scores greater than the set value (set to 4 by default), biomarker with statistical differences between groups, are shown in the histogram of the LDA value distribution. In the evolutionary branching diagram, the circles radiating from inside to outside represent the taxonomic rank from phylum to genus (or species). Each small circle of a different taxonomic level represents a taxon of that level, and the size of the diameter of the small circle is proportional to the relative abundance size.

After 14 days of treatment, it was found by Metastat analysis that *Megamonas* (*p* < 0.05), *Prevotellaceae_UCG_001* (*p* < 0.05), *Ruminococcus_torques_group* (*p* < 0.05), and *Prevotella* (*p* < 0.001) all showed a significant increase in relative abundance, while *Collinsella* (*p* < 0.01), *Muribaculaceae* (*p* < 0.05) showed a significant decrease. The relative abundance of Megamonas, Streptococcus, and Enterococcus was significantly decreased in the EE group compared with the M group (*p* < 0.05). The EE group, in comparison with the K group, significantly increased the relative abundance of *Alistipes* (*p* < 0.05), *Prevotellaceae_UCG_001* (*p* < 0.001), and *Prevotella* (*p* < 0.01). Instead, decreased *Streptococcus*, *Collinsella*, and *Muribaculaceae* in relative abundance (*p* < 0.05) (Figures 4B–D). In the Lefse analysis, it was found that the dominant microbiota in the M group was *Prevotellaceae*, *Prevotellaceae_UCG_001*; the K group mainly had *Streptococcaceae*, *Lactobacilliales*, *Butyricoccus*, *Bacilli* as the dominant microbiota; and the dominant microbiota in the EE group was *Mogibacterium_sp_*, *Prevotella* (Figure 5).

### 3.7. Predicting the metabolic function of microbiota affected by Echinacea

**Figure 6:**
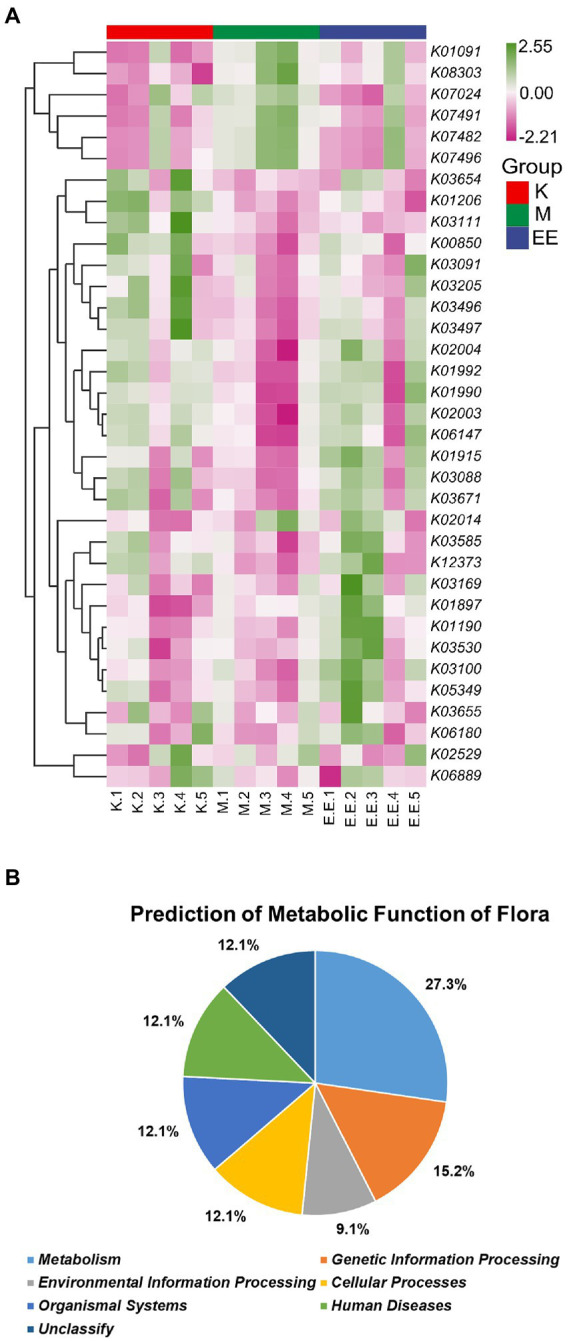
Prediction of metabolic function of the microbiota. **(A)** Heat map of the predicted metabolic functions of the flora based on the KO database (top 35). The horizontal coordinate is the sample name and the vertical coordinate is the associated metabolic pathway number, *N* = 5; **(B)** Plot of the predicted metabolic functions of the flora as a percentage.

Functional predictions based on the KO database showed that among the top 35 metabolic pathways of relevance, the M group had a significantly higher abundance of flora associated with six of these pathways than the K and EE groups, including *K1091*, *K07024*, *K07482*, *K07491*, *K07496*, and *K08303*. Meanwhile the abundance with 15 of these pathways was significantly lower than the other two groups. On the other hand, the EE group had a significantly higher abundance associated with seven of these pathways than the K and M groups, including *K01915*, *K05349*, *K03530*, *K01897*, *K03100*, *K01190*, and *K03169* (Figure 6A). According to the KO database classification of these metabolic pathways, 27.3% of them are related to metabolism, 15.2% to genetic information processing, 12.1% to cellular processes, while organismal systems, human diseases and unclassified each account for 12.1% and environmental information processing for only 9.1% (Figure 6B).

## 4. Discussion

Dexamethasone-induced immunosuppression significantly inhibited the growth performance of ducks. It included a significant slowing of body weight gain, and a marked reduction in the thymus and bursal index (*p* < 0.001). In the trial, immunosuppression damaged the normal structure of the bursa of Fasciola and reduced the number of lymphocytes. And this damage was significantly relieved by the administration of Echinacea extract and restored the number of lymphocytes to a healthy level. The above results indicated that Echinacea extract could effectively repair the damage of the bursa of Fasciola, promote lymphocyte proliferation and improve the immune organ index. It was reported in several studies that the immune-enhancing effects of the polysaccharide components of herbal medicine were mainly achieved by significantly increasing the levels of TNF-α, IFN-γ, and IL-2 in serum (Fan et al., 2013; Zhou et al., 2018; Liu et al., 2022; Nam et al., 2022), and Echinacea extract also increased the levels of these three cytokines in the serum in this trial. It is worth considering that there is a link between changes in serum levels of immune-related cytokines and changes in the intestinal flora. Several studies have reported that the flora is involved in host immunity mainly through their metabolites as signaling factors, acting on immune cells and regulating the expression as well as the release of anti-inflammatory or pro-inflammatory factors. For example, butyric acid in SCFAs can inhibit the proliferation of Th1 cells (Guilloteau et al., 2010), the main cytokines secreted by Th1 cells are TNF-α, IFN-γ, and IL-2, so butyric acid can inhibit the secretion of pro-inflammatory factors and play an immunomodulatory role; or lipopolysaccharide in the flora can promote the secretion of pro-inflammatory factors and induce chronic systemic inflammation (Nicholson et al., 2012).

The intestinal flora, as another organ of the animal body, is not only involved in the digestion and absorption process but also influences the immune process of the animal body by metabolizing and synthesizing essential nutrients needed by the body. The intestinal flora has a crucial role in the development and maturation of the immune system. Lack of colonization of the intestinal flora reduces metabolites associated with the development of the body’s immune organs and tissues, thereby inhibiting the development of the body’s immune function, which may be defective, as is common in germ-free and neonatal animals (Ennamorati et al., 2020; Yang and Cong, 2021). During colonization, infection training enhances the resistance of the microbiota to infection, while stimulating the host immune system to respond, which can promote the development and maturation of the immune system (Butel et al., 2018; Stacy et al., 2021). The absence of specific intestinal flora may affect the maturation and differentiation of immune cells, such as CD4+ T cells in the spleen (Ostman et al., 2006) and Th17 cells in intestinal lymphoid tissue (Ivanov et al., 2009). Conversely, deletion of immune organs can likewise affect the stability of the gut microbiota, as splenectomy can result in abnormal intestinal flora composition in mice (Wei et al., 2021). Immunosuppression can lead to changes in the intestinal flora, which in turn can cause many problems. In this study, Echinacea purpurea was found to regulate the changes in flora caused by immunosuppression.

The sequencing results revealed a recovery trend in the EE group. Although no significant differences were seen in the alpha-diversity indices (*p* > 0.05), the EE group showed an opposite trend in each index compared to the M group, gradually returning to healthy levels. There were similar reports in the intestine of immunosuppressed mice (Fang et al., 2019; Li et al., 2021). Remarkably, immunosuppression significantly increased the abundance of *Megamonas* and *Prevotellaceae_UCG_001*, while *Akkermansia*, *Alistipes*, and *Butyricicoccus* were significantly reduced. In contrast, Echinacea extract is effective in alleviating these changes in flora and may even increase the abundance of beneficial bacteria to improve the immune deficiency of the body. The relative abundance of *Prevotella*、*Prevotellaceae_UCG_001* was significantly higher in the immunosuppressed group. Both could be potential biomarkers in dexamethasone-induced immunosuppression observed in the Lefse analysis. *Prevotella* is strongly associated with systemic and chronic inflammation. *Prevotella copri* may increase the probability of developing colitis by affecting the structure of the flora when colonized in the mouse intestine (Scher et al., 2013). *Prevotella intestinalis* colonization may affect the metabolic processes of the intestinal flora, exacerbating intestinal inflammation and potentially systemic autoimmunity (Iljazovic et al., 2021). Some studies have reported a positive association between *Prevotella* and HIV-induced intestinal inflammation (Dillon et al., 2016). However, further research is needed to uncover the relationship between *Prevotella* and immunosuppression.

*Megamonas*, together with *Bifidobacterium*, can act as beneficial bacteria to regulate the composition of the gut microbiota to promote the synthesis and secretion of SCFAs (Dillon et al., 2016; Wu et al., 2022). In the immunosuppression model group, its elevated abundance may be more associated with the positive aspects. The decrease in its relative abundance correlates with the activation of abnormal immune responses, such as in the intestine of patients with Crohn’s disease (Maldonado-Contreras et al., 2020), immune thrombocytopenia (Yu et al., 2022), or IgA nephropathy (Dong et al., 2020), where its abundance is significantly reduced. It suggests that the relative abundance of *Megamonas* is related to the immune status of the organism. Its abundance increases when the immunity declines, while it decreases significantly with abnormal activation in the immune response.

*Akkermansia* is a genus of beneficial bacteria that has received recent attention in research reports. Its *Akkermansia muciniphila* could enhance the activity of immune cells by being injected intravenously into mice to reduce the tumor burden in mice (Dong et al., 2020; Luo et al., 2021). Its colonization of the intestine increases the expression of genes involved in the immune response, producing IL-8 to participate in the host’s mucosal immune regulation. It also produces mucins that positively works on intestinal epithelial cells to maintain the integrity of the intestinal epithelial mucosa (Derrien et al., 2011; Reunanen et al., 2015). Immunosuppression significantly reduced the relative abundance of *Akkermansia* in the gut to only 0.67% in the immunosuppressed model group. Its low abundance may lead to the absence of the functions described above and put the already immune dysregulated hosts at increased risk of disease infection. However, its relative abundance was significantly higher in the EE group supplemented with Echinacea extract, enhancing the protective effect on the intestine and modulating mucosal immune function. It also displays significant anti-inflammatory properties in the intestine, effectively relieving DSS-induced acute colitis (Qu et al., 2021). Echinacea extract may improve intestinal mucosal immune function and restore host immunity by increasing the abundance of *Akkermansia* in the gut of immunosuppressed ducks. It also enhances the immunity by increasing the abundance of *Alistipes*. Because *Alistipes* could bind to *TLR4* and activate the expression of TNF to enhance the immune clearance of tumor cells (Iida et al., 2013). However, there is no definitive evidence for the main components of Echinacea extract that act with the flora.

The gut microbiota interacts with the host primarily through metabolites produced during the metabolism of the flora. The prediction of the metabolic function of the flora revealed that immunosuppression had a significant effect on the metabolism of the flora and involved metabolic pathways associated with some human diseases and organism systems. While Echinacea extract antagonized the effect of immunosuppression on the mycota and increased the abundance of mycota associated with metabolic functions of human diseases and organic systems. The classification based on the KO database revealed that *KO3671* is associated with the immune system. It has a regulatory role not only in plant immune responses (Mata-Perez and Spoel, 2019) but also in mammals，playing a role in the regulation of immune signal release (Kim et al., 2008; Mougiakakos et al., 2011). It mainly through its protection of cells against oxidation and thus reducing immune cell apoptosis positively affects the immune system (Lu and Holmgren, 2012). *Akkermansia*, *Alistipes*, *Butyricoccus*, and *Bacteroides*, whose relative abundance increased in the EE group, were found to have genes corresponding to *KO3671* in the functional prediction. We speculate that the increased abundance of the genus mentioned above may have increased the *Trx* content in the intestine, exerting its enhancing and modulating effects on the immune system. It could be one of the pathways of immune function modulation by Echinacea extract, but more evidence is needed to prove it.

## 5. Conclusion

To sum up, Echinacea extract can significantly alleviate the immunosuppressive effect of dexamethasone on ducks. It mainly contributes by improving the growth performance of immunosuppressed ducks, restoring the function of immune organs, and regulating the level of immune-related cytokines in the serum. 16 s-rRNA sequencing identified *Prevotella* as a potential biomarker for dexamethasone-induced immunosuppression. Echinacea extract may modulate intestinal immune function by increasing the abundance of beneficial bacterial genera such as *Akkermansia* and *Alistipes* in the intestine. The trial provides a possibility for the application of Echinacea in waterfowl and enriches the research on immunosuppression in waterfowl.

## Data availability statement

The datasets presented in this study can be found in online repositories. The names of the repository/repositories and accession number(s) can be found at: https://www.ncbi.nlm.nih.gov/, PRJNA895924.

## Ethics statement

The animal study was reviewed and approved by the Institutional Animal Welfare and Research Ethics Committee of South China Agricultural University, Guangzhou, China.

## Author contributions

RL, CZ, YS, JC, DG, and SL: were responsible for study conception and design. DS: revised the manuscript. RL, CZ, YS, JC, DG, SL, and DS: were involved in the drafting of the manuscript. All authors contributed to the article and approved the submitted version.

## Funding

The study was supported by the General Project of Guangdong Provincial Natural Science Foundation (2021A1515011010), the Key R&D Project of Guangzhou City (202206010189) and the Project of Young Innovative Talents of Guangdong General Universities (2022KQNCX269).

## Conflict of interest

The authors declare that the research was conducted in the absence of any commercial or financial relationships that could be construed as a potential conflict of interest.

## Publisher’s note

All claims expressed in this article are solely those of the authors and do not necessarily represent those of their affiliated organizations, or those of the publisher, the editors and the reviewers. Any product that may be evaluated in this article, or claim that may be made by its manufacturer, is not guaranteed or endorsed by the publisher.
